# Cerebral Toxoplasmosis As the Initial Presentation of HIV: A Case Series

**DOI:** 10.7759/cureus.23359

**Published:** 2022-03-21

**Authors:** Akshita Khosla, Sachi Singhal, Pooja Jotwani, Robert Kleyman

**Affiliations:** 1 Internal Medicine, Crozer-Chester Medical Center, Upland, USA

**Keywords:** neuropsychiatric manifestations, aids, hiv, cns toxoplasmosis, cerebral toxoplasmosis

## Abstract

The HIV epidemic afflicts millions across the globe, and Sub-Saharan countries bear a disproportionately high burden. Cerebral toxoplasmosis is commonly seen as the disease progresses but is rarely ever reported as the initial manifestation of HIV. The clinical presentation, co-existing risk factors, and outcomes remain underreported. The objective of this article is to report cerebral toxoplasmosis as the initial manifestation of HIV. This is a consecutive series of three patients that presented to a community hospital in Pennsylvania, United States, with a variety of neuropsychiatric symptoms and were found to have cerebral toxoplasmosis. The findings are compared with existing literature on cerebral toxoplasmosis as the initial manifestation of HIV. Cerebral toxoplasmosis as the initial manifestation of HIV is a rarely reported phenomenon. Hyponatremia may be linked with this disease-complex, although further studies are warranted to establish a causal relationship. Co-infection with hepatitis viruses is also a common finding in these patients.

## Introduction

Centers for Disease Control and Prevention (CDC) reports that toxoplasmosis is prevalent worldwide, seen in 11% of the United States (US) population, and can be as high as 60% in tropical areas [1]. There were 36,801 reported new cases of HIV in the US in 2019 with a total of 1,189,700 cases overall [2]. Worldwide, there are roughly 13,138,600 cases of *Toxoplasma gondii *(*T. gondii*) co-infection in people living with HIV (PL-HIV), with 87·1% concentrated in sub-Saharan Africa [3]. Patients with a CD4+ count of less than 100 with seropositivity for toxoplasmosis, have a 30% probability of developing reactivation toxoplasmosis [4]. CNS toxoplasmosis remains the most common cause of cerebral lesions in patients living with AIDS without antibiotic prophylaxis. It is also an important AIDS-defining illness, presenting as the disease progresses without anti-retroviral therapy (ART), with an onus of high morbidity and mortality [5]. Conversely, reported cases of CNS toxoplasmosis as the ﬁrst presentation of individuals infected with HIV/AIDS are exceedingly rare, with only a handful of cases in the literature [6,7,8]. We identified three unique cases presenting to our hospital with neuropsychiatric symptoms. All three were diagnosed with cerebral toxoplasmosis, which subsequently led to the diagnosis of HIV/AIDS.

## Case presentation

Case 1

A 41-year-old African American female, with a past medical history significant for hypertension, presented to our hospital with altered mental status, fevers, and neck pain. She was an immigrant from Liberia. One month prior, she was evaluated in the emergency room (ER) for new-onset myalgias and vomiting and treated for a urinary tract infection. However, the patient returned with progressively worsening confusion. On presentation, she was febrile to 102.1 F, hypertensive to 166/90 mm Hg, and the rest of her vital signs were unremarkable. She had an altered sensorium, was difficult to direct, and mildly agitated. A detailed neurological exam revealed bilaterally reactive pupils, normal in size. No obvious visual field defects were noted, and cranial nerves II-XII were grossly intact. She was moving all four extremities spontaneously and motor strength was noted to be five out of five in each limb, based on the Medical Research Council (MRC) muscle power scale. Patellar reflexes were brisk and symmetric. Gait testing was deferred at that time. Her recent and remote memory as well as attention span were impaired on presentation.

**Figure 1:**
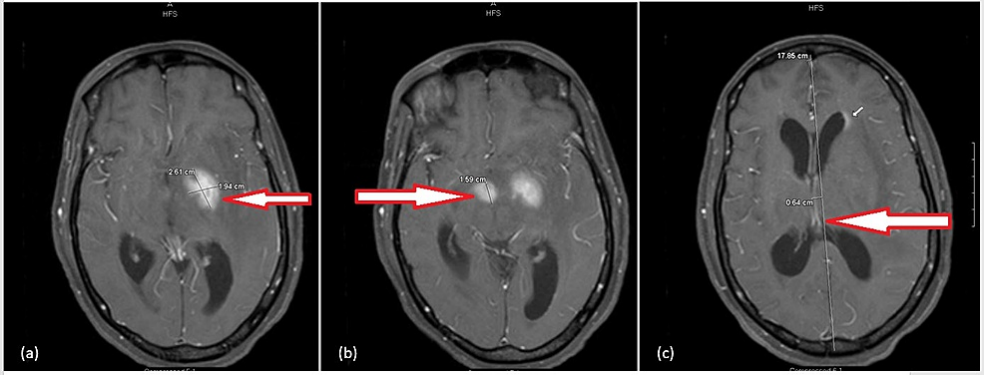
Contrast-enhanced MRI images for Case 1 All sagittal views demonstrating enhancing component of masses: (a) measuring 2.6 x 1.9 x 2.1 cm in the left globus pallidus; (b) measuring 1.7 x 1.6 by 1.0 cm in the hypothalamus; (c) with a 6 mm right to left midline shift

The patient’s blood work revealed sodium (Na) levels of 129 mEq/L with a hemoglobin (Hb) level of 8.7 g/dL. Computed Tomography (CT) scan of the head without contrast demonstrated a hyperdense area in the left basal ganglia with marked swelling and surrounding edema, causing a 7 mm midline shift with mildly dilated ventricles. The differential diagnosis for the patient included malignancies such as glioblastoma, lymphoma, an infectious etiology, or an infarction. The decision to perform a lumbar puncture (LP) was made to evaluate for infectious etiologies. The LP revealed 39 white blood cells, of which 92% were mononuclear. Magnetic Resonance Imaging (MRI) of the brain revealed ring-enhancing masses in several areas of the brain (Figure 1). Findings consistent with obstructive hydrocephalus with a mild left-to-right midline shift were also noted. These findings were suspicious for CNS lymphoma versus cerebral toxoplasmosis. An HIV and hepatitis panel were ordered. Meanwhile, she was initiated on broad-spectrum antibiotics and steroids and *Toxoplasma* IgG titers were sent. The patient had an external ventricular drain (EVD) placed owing to her cerebral edema with mild hydrocephalus. The quantitative *Toxoplasma* IgG titers resulted to be >400 IU/mL. Subsequently, the fourth generation HIV-1/2 Antigen/Antibody combination immunoassay (fourth-generation HIV test), as well as antibodies test for hepatitis C, resulted positive. Her CD4+ count was found to be 50 cells/uL and her viral count for HIV was 301,419 copies/mL. The patient was initiated on a therapeutic dose of trimethoprim/sulfamethoxazole (TMP-SMX), transitioned from intravenous to oral with an improvement of mental status, for a total of six weeks. Two weeks from admission, an ART regimen consisting of bictegravir, emtricitabine, tenofovir was initiated. During her stay, she was also diagnosed with oral *Candida* mucositis for which she finished a course of fluconazole. Repeat MRI obtained after treatment demonstrated significant improvement in the patient's lesions, and the EVD was removed prior to discharge. Through the hospital stay, the patient’s mentation improved remarkably, and she was awake, alert to time, place, and person on day fourteen. Her detailed neurological exam was unrevealing with normal sensory and motor functions on discharge.

Case 2 

A 47-year-old African American female presented to our hospital with bizarre behavior as well as auditory and visual hallucinations. Past medical history was pertinent for hypertension. A majority of the history was obtained from the patient’s siblings who relayed that the patient was originally from Liberia, where she had demonstrated similar behavioral changes, although a formal diagnosis was never established. They also described the patient to be hallucinating at home with insensible speech, disorganized thinking, and behavior. She appeared to be responding to internal stimuli and experiencing hallucinations such as visualizing cars in her living room. She described six people being present in the room when there were only five and claimed to hear a constant knocking inside her head. They also reported changes in her gait, subjective fevers, and chills at home. On presentation, a review of systems could not be elucidated. Upon undertaking a physical exam, she was febrile to 102.7 F and the rest of her vital signs were unremarkable. A neurological examination showed the patient was awake, alert and oriented, and able to follow commands. She had a generalized tremor, which she attributed to chills. Her cranial nerves II-XII were grossly intact and her speech, although clear, was stuttering in nature. Strength in all motor groups of the upper and lower extremities was five out of five based on the MRC muscle power scale. A psychiatric evaluation demonstrated a disoriented and delirious demeanor, with a flat mood and an affect congruent to her mood. Her thought process was deemed illogical and not goal-directed, with loosening of associations. 

**Figure 2:**
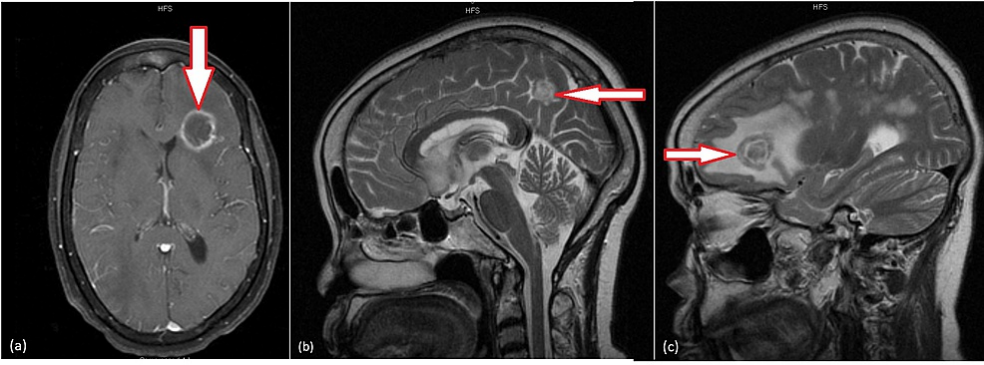
Contrast-enhanced MRI images for Case 2 showing multiple ring-enhancing lesions in the brain, with a large degree of surrounding vasogenic edema. Also observed are concentric alternating zones of hypo- and hyper-intense signals noted on both T2 and T1 sequences, namely “concentric target sign”. (a) and (c) visualize the left frontal lobe lesion sized around 2.5 x 2.1 cm in axial and sagittal views, respectively; (b) is a sagittal view demonstrating a lesion in the right parietal lobe measuring 2.9 x 1.9 cm

The patient’s laboratory work on presentation revealed leukopenia (2.4 x 103/uL), Na levels of 132 mEq/L, and hypokalemia (3.3 mEq/L). CT scan of the head demonstrated extensive edema throughout, and workup was initiated for infectious, malignant, and metabolic etiologies. MRI of the brain showed multiple cerebral ring-enhancing lesions with vasogenic edema and mass effect with no significant midline shift (Figure 2). There arose a strong suspicion for toxoplasmosis versus septic emboli from an unclear source of infection, and appropriate titers and cultures were sent. The day after admission, the patient tested positive for HIV via the fourth generation HIV test. The viral load was 2,427,387 copies/mL and her CD4 count was less than 20 cells/uL. Subsequently, IgG titers for *Toxoplasma* resulted at >400 IU/mL. She also tested positive for hepatitis B surface antigen. She was also diagnosed with *Candida* esophagitis in her hospital stay and managed with fluconazole. The patient was started on a therapeutic dose of TMP-SMX (800-160 mg BID), developed drug rash with eosinophilia and systemic symptoms (DRESS) syndrome resulting in switching over to high dose atovaquone for a minimum of six weeks of therapy. Given her HIV/hepatitis B co-infection, the decision to manage her with emtricitabine, tenofovir, and dolutegravir was made. Serial MRI scans showed progressive improvement of lesions. Within one week, her hallucinations completely resolved. The patient was discharged in a clinically stable condition with a near-total resolution of her presenting symptoms.

Case 3

A 53-year-old African American male, originally from Liberia, presented to our hospital with left leg weakness and difficulty in speaking. Although a poor historian, he was able to express developing difficulty of speech three days prior to this current ED visit. He also complained of right leg weakness seven days ago, which prompted him to visit the ED at the time for evaluation. In his prior visit, his exam was pertinent only for decreased sensation over the right lower leg up to the knee. Cerebellar, cranial nerve, and cognitive function testing were unremarkable on this visit. He was evaluated with a CT of the head, which showed multiple areas of vasogenic edema. This imaging appeared highly suspicious for metastasis. The decision to admit him for further management was made but the patient left against medical advice.

On the current visit, the patient expressed difficulty talking along with left-sided leg weakness. He denied any illicit drug use and had no pertinent medical or surgical history. On examination, his vitals were within normal limits, and he was alert but not oriented, awake but intermittently confused with expressive aphasia. On exam, a right upper extremity pronator drift was noted, but the motor and sensory exam were unremarkable. On cerebellar function testing, right upper extremity dysmetria was also observed on finger-to-nose testing. Cranial nerves II-XII were unremarkable, and gait could not be evaluated. A detailed psychiatric evaluation was unremarkable. Given the patient’s poor recollection of details, his wife was contacted, who reported noticing about a thirty-pound weight loss in the recent past but could not estimate a time frame. She also mentioned a similar self-resolving episode of abnormal speech three months ago. He frequently traveled to Liberia and his last trip was four months prior to his current admission.

**Figure 3:**
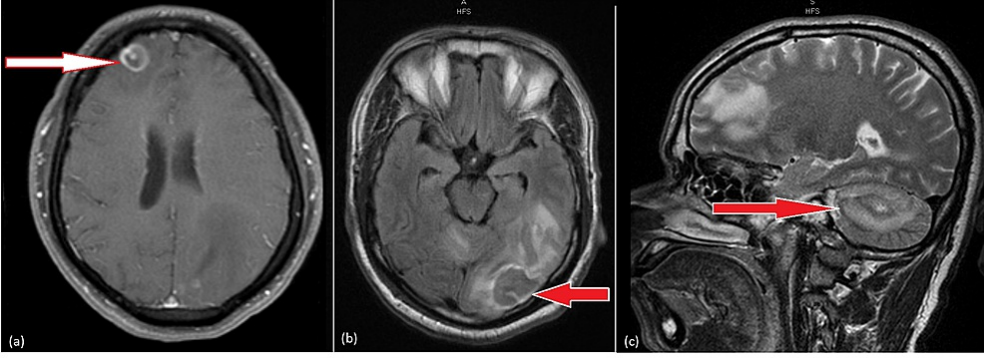
Contrast-enhanced MRI images for Case 3 Numerous ring-enhancing lesions with considerable surrounding vasogenic edema are seen in (a) the right anterior frontal lobe (axial view) measuring 1.3 x 1.3 cm with extensive surrounding edema; (b) the left occipital lobe (axial view) measuring 2.2 x 3.5 cm; (c) the right cerebellar hemisphere (sagittal view) measuring 2.2 x 2.6 cm.

The patient’s laboratory work revealed leucopenia (3.6 x 103/uL) and Na levels of 131 mmol/L. MRI of the brain was performed and demonstrated numerous rim-enhancing lesions with surrounding vasogenic edema and a midline shift to the right (Figure 3) Based on the patient's clinical picture; the differential diagnosis included metastatic disease, or an infectious etiology (including bacterial, mycobacterial or fungal abscesses, toxoplasmosis or neurocysticercosis). The patient was started on dexamethasone to alleviate neurogenic edema, and an initial 4th generation HIV test was negative. CT chest, abdomen, and pelvis to rule out primary malignancy were negative for any processes. During his hospital course, the patient was upgraded to our intensive care unit (ICU) for respiratory distress and lethargy and was eventually intubated. Testing for tuberculosis was sent, T-SPOT.TB immunoassay (Oxford Immunotec, Oxfordshire, United Kingdom) was negative, followed by negative acid-fast staining and cultures. *Toxoplasma* IgG titers were sent but remained pending. Given a high suspicion for malignancy, and negative HIV serology, an open left occipital lobe biopsy was performed. Testing for *Toxoplasma* then resulted, revealing IgG titers >400 IU/mL. Final pathology results from left occipital lobe biopsy had positive immunohistochemistry for *Toxoplasma* and revealed necrotic and inflammatory debris with lymphocytic, neutrophilic, lymphoplasmacytic, and histiocytic infiltrate. Owing to high suspicion of an underlying immunocompromise, repeat HIV combination immunoassay testing was sent ten days after the initial negative HIV test results, which returned as positive. The viral load was 6,624,389 copies/mL at this point, and the CD count was 59 cells/uL. A hepatitis panel showed positive hepatitis A total antibodies (Ig M and IgG). The patient was discharged to a long-term facility on emtricitabine, tenofovir, and dolutegravir for management of his newly diagnosed AIDS, and TMP-SMX for six weeks for management of his toxoplasmosis. On discharge, he had a percutaneous endoscopic gastrostomy (PEG) tube and tracheostomy in place. His pupils were bilaterally reactive and symmetric; he was following commands. His left upper and lower extremity had reduced motor strength of three out of five on the MRC muscle power scale, along with zero out of five MRC muscle power scale noted on the right upper extremity. Only withdrawal to painful stimuli could be elicited in the right lower extremity.

## Discussion

*T. gondii *is an obligate intracellular protozoan parasite with three morphological forms - oocyst (the infective form), tachyzoites, and tissue cysts containing bradyzoites. Infection occurs by ingestion of food or water contaminated by oocytes excreted by cats, or via improperly cooked meat. After ingestion, tachyzoites rapidly multiply and invade nearby cells and disseminate throughout the body via the blood and lymphatics [9]. In immunocompetent individuals, cell-mediated immunity (CMI) is intact and results in a chronic, quiescent life-long infection without eradication. In PL-HIV, opportunistic infection occurs due to depletion of CD4 T-cells resulting in an acute or reactivated infection [10].

Toxoplasmosis is one of the most common infections in humans. It is estimated that about one-third of the global population has latent toxoplasmosis. [11] The prevalence of *T. gondii *infection in PL-HIV follows its prevalence in the general population. In the US, seroprevalence is reported to be about 11-13% [12,13] The prevalence of co-infection in low-income, middle-income, and high-income countries are 55%, 34%, and 26% respectively. Regions with a high prevalence of toxoplasmosis include Latin America, Eastern and Central Europe, the Middle East, and South-East Asia. An exceedingly high burden of disease is borne by the Sub-Saharan African countries [3]. All three of our patients were Liberian in origin, which is in concordance with these studies. 

In individuals infected with HIV/AIDS, toxoplasmosis can present as encephalitis, chorioretinitis, pneumonitis, or as a disseminated disease [13,14,10,15]. The onset of symptoms is subacute with manifestations varying from fevers of unknown origin to a spectrum of neuropsychiatric symptoms [9]. Our patients presented with bizarre behavior and hallucinations and stroke-like symptoms. Differential diagnosis of these characteristic lesions includes CNS lymphoma, progressive multifocal encephalopathy (PML), cerebral tuberculosis (CNS-TB), focal *Cryptococcus* infection, cytomegalovirus, and bacterial brain abscess. It is exceedingly difficult to differentiate CNS lymphoma from CNS toxoplasmosis given overlapping clinical features and imaging, but negative serologies for toxoplasma and detection of Epstein-Barr Virus DNA in cerebrospinal fluid (CSF) assist in arriving at the diagnosis [16]. Other differentials can also be excluded based on blood and cerebrospinal fluid testing [17]. Details regarding neuroimaging of our patients are outlined in Table 1.

**Table 1:** Summary of presentation, management, and outcomes of previously reported cases and this case series ND: not disclosed; AMS: altered mental status; TMP-SMX: trimethoprim-sulfamethoxazole; GTCS: generalized tonic-clonic seizure; CA: current article

Case	Age (Years)	Sex	Country of origin	Presenting complaint	CD4+ Count on diagnosis (cells/mm^3^)	Viral load on diagnosis (copies/mL)	CT scan findings	MRI findings	Co-infection with hepatitis	Hyponatremia (mEq/L)	Antimicrobial therapy	HAART
Abbasi Fard et al., 2020 [6]	9	Male	ND	AMS, GTCS, fever	<100	ND	ND	Diffuse ring-enhancing lesions with perifocal edema and significant midline shift	ND	ND	TMP-SMX	Not started as patient passed away on day 6 of admission
Barman et al., 2018 [7]	62	Male	ND	Low-grade fever, pruritic rash of upper extremities, and chest wall	<100	ND	ND	Multiple ring-enhancing lesions with perilesional edema in the right thalamus, left cerebellar, and bilateral cerebral hemisphere	No	ND	Sulfadiazine, pyrimethamine, clindamycin	ND
Zoubi et al. 2017 [8]	39	Male	Venezuela	Behavioral disturbances, headache, nausea, and chills	14	34,913	ND	Ring-enhancing lesion in the right basal ganglia with central area of necrosis and extensive surrounding vasogenic edema causing a 7 mm midline shift to the left	ND	ND	TMP-SMX six weeks	Elvitegravir, cobicistat, emtricitabine, Tenofovir
Case 1, CA, 2022	41	Female	Liberia	AMS, fever and neck pain	50	301,419	Hyper dense area in the left basal ganglia with marked swelling and surrounding deep white matter edema. This lesion caused a 7 mm midline shift with mildly dilated ventricles	Ring enhancing masses in the left globus pallidus and hypothalamus with extensive surrounding vasogenic edema, with obstructive hydrocephalus, and a mild left-to-right midline shift​	Hepatitis C	129	TMP-SMX six weeks	Bictegravir, emtricitabine, tenofovir
Case 2, CA, 2022	47	Female	Liberia	AMS, bizarre behavior, auditory and visual hallucinations	<20	2,427,387	Multiple cerebral ring-enhancing lesions with vasogenic edema and mass effect	Multiple cerebral ring-enhancing lesions with vasogenic edema and mass effect with no significant midline shift	Hepatitis B	132	Atovaquone six weeks	Emtricitabine, tenofovir
Case 3, CA, 2022	53	Male	Liberia	Left leg weakness and difficulty speaking	59	624,389	Vasogenic edema in the right frontal lobe, right cerebellar hemisphere, and a larger area of vasogenic edema in the left posterior parietal, temporal and occipital lobes, as well as within left posterior corpus callosum	Numerous rim enhancing lesions with considerable surrounding vasogenic edema throughout the brain (at least 20 were noted). There was also a mild resultant midline shift to the right by three millimeters	Hepatitis A	131	TMP-SMX six weeks	Emtricitabine, tenofovir, dolutegravir

Apart from HIV, CNS toxoplasmosis is seen in other immunocompromised states like X-linked hyper-IgM syndrome [18], common variable immunodeficiency (CVID) [19], and patients with a history of heart [20], liver [21], or hematopoietic stem cell transplants [22,23].

CNS toxoplasmosis is an AIDS-defining illness, and almost always occurs as the disease progresses. CD4+ counts are often less than 100 cells/uL. However, it is exceedingly rare for cerebral toxoplasmosis to be the initial manifestation of HIV/AIDS. Three cases have been reported thus far with each case leading to the diagnosis of disseminated cerebral toxoplasmosis as the initial presentation of HIV/AIDS [6,7]. Further details about the presentation, management, and outcomes of these cases are summarized along with our cases in Table 1.

Of note, case number three in our case series was initially deemed HIV negative, but a high suspicion prompted repeat testing, which was then positive. The current standard of care in our hospital is a fourth-generation Antigen/Antibody combination assay, which has a sensitivity of 100% and a specificity of 96.7-97.8% [24,25]. The median window for this test is 18 days (IQR of 16-24) [26]. Although this finding was highly suggestive of an acute infection with HIV, there is a high probability that it was a false-negative test result tested during the window period. However, the case still reiterates that toxoplasmosis in this instance was the first presentation of AIDS. 

Hyponatremia has been previously reported in conjunction with CNS toxoplasmosis. Duzovali et al. reported isolated hyponatremia as the only initial manifestation of cerebral toxoplasmosis, with the recovery of Na levels when placed on appropriate antibiotics [27]. A retrospective study curtailing 92 patients with toxoplasmic encephalitis (TE) demonstrated low Na levels in significant correlation with increased mortality [28]. In another series of 259 patients with AIDS, hyponatremia was related to increased mortality and length of stay [29]. Plausible underlying mechanisms include cerebral salt wasting syndrome, increased gastrointestinal losses, adrenal insufficiency, and syndrome of inappropriate anti-diuretic hormone (SIADH) from CNS involvement [30]. Hyponatremia was noted in all three of our patients on admission, and further research is needed to explore the correlation and diagnostic value in the evaluation of patients with cerebral toxoplasmosis with and without HIV.

The timing of initiation of highly active antiretroviral therapy (HAART) is another key point of discussion. Early initiation of HAART in patients with cerebral toxoplasmosis may lead to the development of toxoplasmic encephalitis immune reconstitution inflammatory syndrome (TE-IRIS) [31,32]. This may lead to the unmasking of a previously unknown infection or a paradoxical worsening of a known infection in patients on appropriate antimicrobial therapies. It has been demonstrated that delayed initiation of HAART timed to two weeks after antibiotic management is prudent. This practice was followed in all three of our patients [33].

In addition to appropriate antibiotics and delayed HAART initiation, a comprehensive physical evaluation must be done for these patients as they are at risk of developing other opportunistic infections. The hospital course of two of our three patients was complicated with *Candida *infections.

Lastly, PL-HIV have a marked rate of co-infection with other blood-borne pathogens, especially hepatitis B and C. In China, the seroprevalence of hepatitis B and hepatitis C concomitant to HIV was noted to be up to 13.7% and 24.7%, respectively [34]. Globally, the co-prevalence of hepatitis B and HIV is estimated to be at 7.6%, with a prominent percentage of these cases being concentrated in Sub-Saharan Africa (69% of the global burden) [35]. Although mainly seen with ingestion of contaminated food and international travel, hepatitis A is also transmissible via sexual contact, illicit drug use, and blood transfusions [36]. However, data pertaining to co-infection rates of hepatitis A and HIV is lacking in the existing literature. A new diagnosis of HIV should prompt evaluation and testing for other blood-borne pathogens as it serves as a guide to anti-retroviral therapy, and co-infection in and of itself is related to accelerated liver injury. Interestingly, all three of our patients were co-infected with hepatitis viruses, and HAART was tailored accordingly. Of note, patients co-infected with hepatitis B and HIV should receive tenofovir and emtricitabine-based regimens owing to their high efficacy against both viruses [37].

## Conclusions

This case series highlights some astute observations regarding the initial finding of CNS toxoplasmosis that led to a diagnosis of HIV. We observed several common themes between the cases such as presentation of mild hyponatremia, similar demographics, presence of other opportunistic infections, and coinfections with hepatitis B and C that warranted a tailored therapy. The manifestations of CNS toxoplasmosis are spread across a wide spectrum, and this is an important differential in a patient with nonspecific neuropsychiatric symptoms. People living in the Sub-Saharan region bear a disproportionate share of the disease burden. Factors such as delay in receiving care, limited resources, or limited health education may play a role in unmasking of HIV in this delayed fashion. 
